# Evaluation of the ability of standardized supports to improve public health response to syndromic surveillance for respiratory diseases in Canada

**DOI:** 10.1186/s12889-017-4073-6

**Published:** 2017-02-15

**Authors:** Laura A. Rivera, Ye Li, Rachel D. Savage, Natasha S. Crowcroft, Shelly Bolotin, Laura C. Rosella, Wendy Lou, Jessica Hopkins, Ian Gemmill, Ian Johnson

**Affiliations:** 1 Public Health Ontario, 480 University Ave, Toronto, M5G1V2 Canada; 2 Dalla Lana School of Public Health, University of Toronto, 155 College Street, Toronto, M5T 1P8 Canada; 3 City of Hamilton Public Health Services, 71 Main Street West, Hamilton, Ontario L8P 4Y5 Canada; 4 Department of Clinical Epidemiology and Biostatistics, McMaster University, 1280 Main Street West, Hamilton, L8S 4K1 Canada; 5 KFL&A Public Health, 221 Portsmouth Avenue, Kingston, K7M 1V5 Canada

## Abstract

**Background:**

Despite widespread implementation of syndromic surveillance systems within public health agencies, previous studies of the implementation and use of these systems have indicated that the functions and responses taken in response to syndromic surveillance data vary widely according to local context and preferences. The objective of the Syndromic Surveillance Evaluation Study was to develop and implement standardized supports in local public health agencies in Ontario, Canada, and evaluate the ability of these supports to affect actions taken as part of public health communicable disease control programs.

**Methods:**

Local public health agencies (LPHA) in Ontario, which used syndromic surveillance based on emergency department visits for respiratory disease, were recruited and randomly allocated to the study intervention or control group. The intervention group health agencies received standardized supports in terms of a standardized aberrant event detection algorithm and a response protocol dictating steps to investigate and assess the public health significance of syndromic surveillance alerts. The control group continued with their pre-existing syndromic surveillance infrastructure and processes. Outcomes were assessed using logbooks, which collected quantitative and qualitative information about alerts received, investigation steps taken, and public health responses. The study was conducted prospectively for 15 months (October 2013 to February 2015).

**Results:**

Fifteen LPHAs participated in the study (*n* = 9 intervention group, *n* = 6 control group). A total of 1,969 syndromic surveillance alerts were received by all LPHAs. Variations in the types and amount of responses varied by LPHA, in particularly differences were noted by the size of the health unit. Smaller health units had more challenges to both detect and mount a response to any alerts. LPHAs in the control group were more likely to declare alerts to have public health significance and to initiate any action. Regression models using repeated measures showed an interaction between the year (Year 1 versus Year 2) and the intervention as well as an interaction between year and sustained nature of the alert. Both of these were linked to the control health units reporting more “watchful waiting”.

**Conclusions:**

This study raises questions about the effectiveness of using standardized protocols to improve the performance of syndromic surveillance in a decentralized public health system. Despite efforts to create standardized protocols and engage public health agencies in the process, no significant differences in the effective use of syndromic alerts were observed beyond year 1. It also raises questions about the minimum capacity of the agency and minimum population size that are required for an effective response.

## Background

Syndromic surveillance, defined as the use of pre-diagnostic data for surveillance purposes, has risen in prominence and acceptance as part of standard practice in public health surveillance [1, 2]. Despite some early detractors [3, 4], implementation of these systems began to gain traction from the introduction of anti-bioterrorism public health programming in the 2000s [5–7]. While the originally intended functions of syndromic surveillance were to support early detection and response to communicable disease outbreaks, their utility has expanded since that time to support situational awareness, to provide value to public health staff in having a “continuously acquired” data source of pre-diagnostic information, to provide reassurance that an outbreak or aberrant event is not occurring particularly during mass gatherings, and to augment existing surveillance infrastructure [8, 9]. Many of these roles are relevant to non-communicable as well as communicable disease surveillance.

While the span and scope of syndromic surveillance has expanded since its inception, evaluations of syndromic surveillance have focused on syndromic surveillance systems’ ability to detect known or simulated communicable disease outbreaks [10–12]. Due to the challenges of determining an appropriate level of sensitivity and specificity of aberration detection algorithms, considerable effort has also been placed on such optimization of statistical algorithms [13]. In comparison, we note that few studies have prospectively assessed the impact of syndromic surveillance on outcomes beyond outbreak detection, and even fewer have investigated how syndromic surveillance systems for infectious diseases are employed in local public health professional practice.

Studies on the experiences of using syndromic surveillance systems to inform public health actions have shown high uptake but wide variation in application. In the United States, studies have shown that between 80 and 94% of state health departments have at least one syndromic surveillance system in operation but there was variation in terms of the data sources used [14, 15]. In addition, some states had centralized systems while others were decentralized [14, 15]. In Ontario, Canada, 53% (20/38) of public health organizations use syndromic surveillance systems for infectious disease surveillance [16]. Subsequent work in Ontario, which assessed the use of syndromic surveillance systems during the 2009 H1N1 influenza pandemic, demonstrated a wide variation in the uses, procedures, and corresponding public health actions in response to syndromic alerts [17, 18] in this decentralized system.

The objective of this study was to determine whether the development and implementation of standardized supports, consisting of a standardized method for detecting aberrant events and a standardized response protocol for handling such events, were able to support more effective use of syndromic surveillance emergency department data for infectious respiratory diseases compared to usual practices.

## Methods

### Setting and study participants

In Ontario, Canada, there are 36 local public health agencies (LPHAs) that each provides public health programming for a distinct jurisdiction. The populations served vary widely between over 2 million residents in the largest agency to under 200,000 residents in the smallest. While Ontario LPHAs must meet standards for providing public health services, including infectious and communicable disease control [19], the use of syndromic surveillance is not a provincial requirement. Each health agency independently decides if they wish to implement syndromic surveillance systems. Of the possible syndromic data sources, syndromes based on aggregated presenting complaints to emergency departments are most commonly used [20, 21]. One such system, called the ‘Acute Care Enhanced Surveillance System’ (ACES), is used by the majority of Ontario LPHAs, but some agencies administer their own systems [22].

### Study design, participant recruitment, and experimental allocation

This study takes a cluster randomized study design, where the level of study participant is the Ontario LPHA. LPHAs eligible to participate were those that self-identified as regular users of emergency department syndromic surveillance systems within their communicable disease programs. We recruited study participants by providing each agency’s Medical Officers of Health with an information letter and invitation to participate. Next, consenting LPHAs were allocated to either the intervention or control arms of the study. In order to balance the intervention and control groups to contain health units with a similar distribution of population sizes served, LPHAs were ranked by size of the population served, alternately assigned a code of “0” or “1”. Based on a coin flip, agencies in the “0” group were assigned to be the control arm, and those in the “1” group assigned the intervention group.

### Study interventions: development and implementation of the study algorithm and protocol

The study team developed two standardized support interventions: (1) a standard aberration detection algorithm for application to emergency department visit data, and (2) a standard response protocol to guide communicable disease staff in investigating and acting on syndromic surveillance alerts. The interventions were developed in a participatory fashion where the research team received significant input from the local communicable disease staff throughout the process [23].

To develop the standardized aberration detection algorithm, we acquired 3 years of historical emergency room visit data for respiratory syndrome and influenza-like-illness from each LPHA. Based on these data, a linear regression model was developed to adjust for seasonal factors as well as factors such as the day of the week. The cumulative sum (CUSUM) method applied to differences between the observed number of visits and that predicted by the regression model. [24, 25]. The study team held a face-to face meeting of representatives from the intervention arm health agencies, in order to choose the best model and elicit feedback regarding the algorithms’ relative sensitivity and specificity. As a result of this exercise, we determined that the study participants preferred the algorithm to be more specific than sensitive. More details on the algorithm development can be found in Appendix.

The second component of the study intervention consisted of developing and implementing a protocol to guide health agency communicable disease staff in handling and acting upon syndromic surveillance events. The protocol was based on the findings from a review of peer-reviewed and gray literature. Two of the most relevant documents were a study by Usher-Pines et al. [26] and the results of a consensus document [27]. A face-to-face meeting of representatives from the intervention LPHAs was used to reach consensus on the components of the response protocol. The protocol consisted of 3 phases: (1) checking whether the syndromic alert could be attributed to an alternate explanation, such as data misclassification, missing or duplicated data, etc., (2) validating the alert epidemiologically by assessing clustering by person, place, and time, and (3) assessing the public health significance of the syndromic alert in the context of other information.

### Intervention implementation

The standardized supports were implemented in intervention LPHAs between October 2013 and February 2015. During this period, LPHAs in the intervention group: (1) received additional weekly reports of alerts generated by the standardized aberration detection algorithm applied to the data for the respective LPHA, and (2) were asked to apply the steps outlined in the standard protocol for investigating all syndromic alerts received. Due to feasibility issues regarding data transfer to the research team who applied the standard aberration detection algorithm, study-generated syndromic alerts were approximately one week late in being disseminated to the intervention LPHAs. Since we had no evidence that the study-generated algorithm was more effective than each LPHA’s existing algorithms, intervention arm health agencies were instructed to continue to receive alerts from existing syndromic surveillance systems. Meanwhile, control LPHAs continued with their usual practices in regards to receiving, investigating and responding to syndromic alerts from their existing syndromic surveillance systems for detecting aberrant events.

Given the nature of the intervention, blinding was not possible. In order to minimize contamination, intervention LPHA staff were asked not to share details pertaining to the study-generated algorithms or about the response protocols with public health colleagues in control LPHAs.

### Data sources and study variables

All emergency department data systems relied on triage diagnoses provided at the time of patient registration at the emergency department. While ACES is used by most LPHAs, a few LPHAs have their own systems. Coding of the respiratory and ILI syndromes was assessed and, while not exactly the same, were felt to be sufficiently similar.

As our primary data collection mechanism, we used logbooks which were completed by relevant LPHA staff. The logbooks consisted of pre-programmed Excel worksheets that collected information about syndromic surveillance alerts received by LPHAs. For each alert, the LPHA staff recorded the investigative steps taken and any public health responses initiated. The logbooks contained two categorical fields (i.e. “Was a public health response warranted?” and “Did your response lead to detection of an outbreak?”) and multiple free-text fields. The latter provided narrative comments on the investigative steps and actions taken. At the end of the logbook data collection period, we held semi-structured interviews by telephone with each of the participating LPHAs’ study contacts in order to validate information in the logbooks, to verify the absence of study contamination, and to gain a better understanding of the reasons for some of the reported responses and non-responses. The interviews were recorded and transcribed verbatim, forming a qualitative data source to corroborate the information provided in the logbooks.

The outcome measures of “effective use” of syndromic surveillance data were initially defined in terms of two categorical variables. These were the (1) number of alerts that were perceived to warrant a public health response, and (2) the number of alerts that led to the detection of an outbreak or signalled the start of the influenza season. The research team also coded the text fields in order to create the appropriate outcome variables. These outcomes were: (A) the number of alerts with results communicated internally within the LPHA, (B) the number of alerts with results communicated to external entities such as hospitals, and (C) “watchful waiting”, defined as reassessment of syndromic alert results the following day. “Watchful waiting” was not originally included as a valid outcome. However, since many LPHA’s reported “watchful waiting” as an action, it was included in the analysis. Upon preliminary analysis of the logbooks, it became apparent that the variable “perceived to warrant a public health response” was often missing and frequently not related to what was listed in the open text fields. Following-up interviews with the LPHAs revealed that the open text fields more accurately reflected their actions.

The primary independent variables were: the allocation status of the LPHA to the study intervention or control group, the “size of the LPHA according to population served” (categories: large health unit, population > 400,000; medium health unit, population between 150,000 and 399,999; and small health unit, population less than 150,000), “nature of alert with respect to being new or sustained” (categories: “new alert”, characterizing an alert whose results had not previously been seen; “sustained alert”, characterizing an alert whose results were felt to be related to an alert received previously). Small LPHAs were more likely to rely on one hospital as their reporting source and have number of visits per day to each hospital. They also have staff providing only part-time support to the syndromic surveillance program. Post-hoc predictor variables that were felt to be important upon preliminary logbook analysis were: “year of the study” (categories: Year 1, October 14, 2013- August 30, 2014; Year 2, September 1, 2014 - February 27, 2015), as well as whether or not the LPHA’s communicable disease staff perceived that syndromic data was a primary source of surveillance information to inform public health action (categories: yes, no).

The final dataset contained the categorical information captured in the logbooks (outbreak detected (yes/no)), additional numerical variables derived from inductive thematic analysis of the free-text fields within the logbooks themselves, as well as variable from the inductive thematic analysis of concepts that arose in semi-structured interviews (i.e. whether the LPHA considered syndromic surveillance primary) [28, 29].

### Data analysis

We generated descriptive statistics to characterize syndromic alerts received by the LPHAs, as well as corresponding investigative steps and public health actions taken, describing categorical variables using frequencies and proportions.

Due to each LPHA replicating the same internal process for each alert, no statistical tests were applied to the descriptive results. Accordingly, logistic regression models with random effects accounting for repeated observations from LPHAs were used in order to assess the impact of the independent variables on the outcomes. Variables included in the regression were based on a conceptual model regardless of their statistical significance on the outcome. Since the effect of the small and medium public health authorities may be different, alerts from each were each coded as separate dummy variables with the large LPHA was used as the reference point. Similarly, new alerts were coded as the reference point for sustained alerts.

### Study protocol modifications

The study was intended to run from October 2013 to August 2014. However, a severe ice storm struck Ontario at the end of December 2013 and, during this time, syndromic surveillance reporting systems were disrupted. While the data were retrospectively populated in the system, the potential for a real-time response to alerts was lost. This storm also happened to coincide with the peak of Influenza A activity in 2013 [30]. As a result, all participating public health units were asked to extend the study for an additional five months until the end of February 2015. Only one LPHA declined to participate in this extension but its data are included in the year 1 analysis. During this extended phase (Year 2) of the study, Ontario experienced a severe influenza season where the vaccine was shown to have low effectiveness [31].

## Results

### Study participants

Random allocation of the 16 participating LPHAs resulted in 9 LPHAs in the intervention group and 6 LPHAs in the control group (Table 1). The original allocation was 8 and 7. However, since one control LPHA conducted only school absenteeism surveillance, it was excluded from this portion of the study. Finally, one LPHA volunteered after the initial allocation had occurred, and was assigned randomly separately. Based on this coin flip, it was assigned to the intervention group.

**Table 1:** Characterization of Ontario public health units recruited to participate in the study

	Total study population (n, health units)	Intervention group (n, health units)	Control group (n of health units)
Population served by the LPHAs			
Large (Population > 400,000)	7	4	3
Medium (Population 150,000 to 399,999)	5	3	2
Small (Population < 150,000	3	2	1
Emergency department syndromic system			
ACES system	13	9	4
LPHA specific system	2	0	2
Total	15	9	6

Thirteen (13) LHPAs used data from the ACES emergency department visit syndromic surveillance system [31], while two health units had LPHA specific systems (Table 1). By chance, the two LPHAs with their own detection systems were allocated by chance to the control group.

### Dataset development via inductive thematic analysis

To develop the dataset that was used for regression analysis, inductive thematic analysis of the logbook data was necessary to extract key themes that were subsequently used to derive numerical variables [28]. Two authors (LAR, RDS) independently coded logbook data received for October 2013 to June 2014, and compared results. Disagreements were resolved via discussion with a third author (ILJ). The Kappa score for inter-observer agreement was 0.70. All data following June 2014 were coded by one author (LAR).

### Characterization of aberrant events received by public health unit study participants

A total of 1,969 alerts were included in the study from October 2013 to February 2015. The control LPHAs reported receiving 1,027 alerts while the intervention LPHAs reported receiving 942 alerts (Table 2). Of the total, 789 were for respiratory syndrome, and 1,180 were for the influenza-like illness (ILI) syndrome. Five hundred and twenty eight alerts (26%), were classified as new alerts while 1,431 (73%) were classified as sustained. Fifty nine percent (59%) of alerts were seen in study Year 2, between September 2014 and February 2015 (*n* = 1,168). Two hundred and twelve (11%) alerts were generated by the study-developed standard algorithm.

**Table 2:** Characterization of the investigative activities taken regarding syndromic alerts, serving as a process evaluation measure

	Total (n, alerts)	Intervention group (n, alerts)	Control group (n, alerts)
Alerts received by local public health agency study participants	1969	942	1027
Year 1	801	409	392
Year 2	1168	533	635
Alerts checked for data misclassification, data stream issues, or error-related issues	305 (15%)	224 (24%)	81 (8%)
Year 1	193 (24%)	156 (38%)	37 (9%)
Year 2	112 (10%)	68 (13%)	44 (7%)
Alerts checked for epidemiological clustering	1538 (78%)	664 (70%)	874 (85%)
Year 1	635 (79%)	253 (62%)	382 (97%)
Year 2	903 (77%)	411 (77%)	492 (77%)
Alerts warranting public health action	714 (36%)	165 (18%)	549 (53%)
Year 1	319 (40%)	11 (3%)	308 (79%)
Year 2	395 (34%)	154 (29%)	241 (38%)
Missing	380 (19%)	168 (18%)	212 (21%)

### Steps taken to investigate syndromic surveillance aberrant events

In order to assess the degree to which the study-developed standard response protocol was implemented in the LPHAs, the steps taken in the investigation of each alert were assessed in both the intervention and control LPHAs. Intervention LPHAs reported a higher frequency of assessing syndromic alerts for alternative explanations compared to control LPHAs, 24% of the alerts compared to 8% respectively (Table 2). This effect varied by the size of LPHA, where those serving a larger population (population size > 400,000) more frequently looked for alternative explanations. The proportions for medium and small-sized health agencies and in year 2 of the study were similar to the proportions observed in the control LPHAs. All LPHAs were more likely to look for alternative explanations in new alerts than sustained alerts.

Control LPHAs were more likely to investigate the alerts for epidemiological clustering by assessing for temporal, spatial, and demographic trends. The proportion of alerts being investigated for epidemiological clustering was relatively constant across all control LPHAs, but the proportion declined from approximately 80% among Intervention LPHAs serving large populations to 55% those serving a small population.

### LPHA responses to syndromic surveillance aberrant events

Information on the public health significance of an alert was difficult to assess, since 19% of the responses were missing. For 16% of instances where a response was reported as being warranted, no action was reported in the free text field. Similarly for situations where a public health response was indicated as not warranted, 38% reported a public health action in the free text fields. Based on these challenges, this variable was not used in further analyses.

**Fig. 1:**
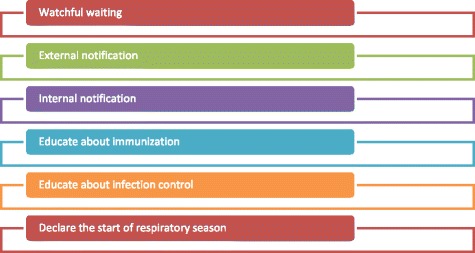
Illustration of the breadth of public health actions taken in response to emergency department respiratory syndromic visit alerts by Ontario public health units, October 2013-February 2015

Logbook thematic analysis indicated that syndromic alerts informed a breadth of public health actions, illustrated in Fig. 1. Using these definitions, there were 946 (48%) instances reported overall of any public health action being taken, with 382 (41% of all alerts) for the intervention LPHAs and 564 (55% of all alerts) for control LPHAs. The number of responses was lowest in the intervention LPHAs in year 1 of the study, between October 2013 and August 2014 (Table 3).

**Table 3:** Characterization of the types of responses initiated regarding 545 syndromic alerts, serving as an outcome evaluation measure

	Total (n, alerts)	Intervention group (n, alerts)	Control group (n, alerts)
Number of alerts	1,969	942	1,027
Year 1	801	409	392
Year 2	1,168	533	625
Any action	946 (48%)	382 (41%)	564 (55%)
Year 1	397 (50%)	74 (18%)	323 (82%)
Year 2	549 (47%)	308 (58%)	241 (38%)
Watchful waiting	647 (33%)	263 (28%)	384 (37%)
Year 1	275 (34%)	18 (4%)	257 (66%)
Year 2	372 (32%)	245 (46%)	127 (20%)
Internal notification	297 (15%)	146 (15%)	151 (15%)
Year 1	109 (14%)	55 (13%)	54 (14%)
Year2	188 (16%)	91 (17%)	97 (15%)
External notification	171 (9%)	84 (9%)	87 (8%)
Year 1	66 (8%)	5 (1%)	61 (16%)
Year 2	105 (9%)	79 (15%)	26 (4%)

In terms of outbreak detection, there were no outbreaks reported as detected by alerts from syndromic surveillance during the study period. Two LPHAs, with one in the intervention group and one in the control group, reported that syndromic alerts helped reassure them that the influenza season had begun but the actual decision was made using other data (a combination of school absenteeism syndromic surveillance and laboratory testing for influenza).

Of the reported public health responses in response to syndromic alerts, the most common action was “watchful waiting”. This activity corresponded to approximately one third of all the alerts and two thirds of the reported actions taken. The other responses of providing internal notification within the health agency regarding details about the syndromic alert (“notifying internally”), or sharing details about the syndromic alert with external groups such as hospital partners (“notifying externally”) were much lower at 15 and 9% respectively. No difference was observed between study generated alerts and those generated by their systems.

### Predictors of outcomes related to public health responses to syndromic alerts

Logistic regression models confirmed the descriptive results, where control LPHAs reported significantly more overall responses even when the models adjusted for population size served by the LPHA. The analysis also showed an interaction between the year (Year 1 versus Year 2) and the intervention as well as an interaction between year and sustained nature of the alert. As a result, Table 4 shows the regression models by year. The effect of the intervention was more pronounced in year 1 with large LPHAs in the intervention group showing fewer responses to the alerts than control LPHAs. This effect was less in year 2. In year 1, LPHAs were more likely to respond to sustained alerts but this effect was reversed in year 2, mainly due to the increase in the “watchful waiting” response to the first alert. No significant results were seen for the medium or small LPHAs.

**Table 4:** Multivariable logistic regression models that account for repeated measures assessing the association between specified predictor variables and likelihood of taking a public health response to emergency department syndromic surveillance alerts by year within the study

		Outcomes				
		(Odds Ratio, 95% Confidence Interval)				
		Any response	Any response excluding watchful waiting	Watchful waiting	Internal notification	External notification
Intervention	Year 1	0.024 (<0.01, 0.51)^a^	0.43 (0.06, 2.92)	0.04 (0.001, 1.58)	2.94 (0.12, 72.2)	0.04 (<0.01, 0.37)^a^
	Year 2	0.20 (0.02, 2.38)	0.69 (0.16, 3.03)	0.19 (<0.01, 55.8)	0.98 (0.08, 12.4)	1.27 (0.19, 8.34)
Control		Reference	Reference	Reference	Reference	Reference
Medium LPHA	Year 1	0.83 (0.15, 45.9)	0.91 (0.06, 1.39)	4.25 (0.04, 423)	0.35 (0.01, 21.2)	3.66 (0.18, 74.0)
	Year 2	0.29 (0.012, 7.12)	0.45 (0.06, 3.2)	11.7 (0.009, 15,000)	0.78 (0.04, 17.5)	1.18 (0.10, 13.9)
Large LPHA		Reference	Reference	Reference	Reference	Reference
Small LPHA	Year 1	0.352 (0.13, 9.30)	1.02 (0.12, 8.44)	1.28 (0.02, 69.2)	0.61 (0.02, 15.9)	3.83 (0.29, 50.5)
	Year 2	1.65 (0.11, 24.0)	0.56 (0.11, 2.86)	32.1 (0.05, 19,908)	0.19 (0.01, 3.33)	1.62 (0.21, 12.6)
Large LPHA		Reference	Reference	Reference	Reference	Reference
Sustained alert	Year 1	1.81 (1.10, 2.99)^a^	2.06 (1.23, 3.45)^a^	0.62 (0.24, 1.56)	2.21 (1.27, 3.84)^a^	7.83 (3.2, 19.2)^a^
	Year 2	0.24 (0.16, 0.35)^a^	0.92 (0.61, 1.39)	0.15 (0.10, 0.23)^a^	0.75 (0.47, 1.19)	0.59 (0.37, 0.94)^a^
New alert		Reference	Reference	Reference	Reference	Reference

^a^Statistically significant result

The post-hoc-generated logistic regression model that included a variable for whether LPHA staff perceived syndromic data as a primary source of information showed that those agencies who perceive syndromic surveillance as a primary source of data were much more likely to report any type of response to an alert in year 1 of the study but not year 2. However, when the term for “watchful waiting” is excluded from public health response, the results were not significant (Table 5).

**Table 5:** Post-hoc multivariable logistic regression models that account for repeated measures assessing the association between specified predictor variables, including the variable of whether or not syndromic surveillance was a primary data source on outcomes related to the likelihood of taking a public health response to emergency department syndromic surveillance alerts by year within the study

		Outcomes	
		(Odds Ratio, 95% Confidence Interval)	
		Any response including watchful waiting	Any response excluding watchful waiting
Intervention	Year 1	0.020 (<0.01, 0.18)^a^	0.41 (0.06, 2.78)
	Year 2	0.18 (0.016, 2.12)	0.72 (0.18, 3.00)
Control		Reference	Reference
Medium LPHA	Year 1	0.38 (0.02, 7.33)	0.79 (0.06, 10.2)
	Year 2	0.21 (0.01, 5.41)	0.52 (0.08, 3.44)
Large LPHA		Reference	Reference
Small LPHA	Year 1	1.12 (0.01, 12.7)	1.30 (0.14, 11.9)
	Year 2	2.49 (0.16, 39.1)	0.47 (0.09, 2.43)
Large LPHA		Reference	Reference
Sustained alert	Year 1	1.87 (1.13,3.08)^a^	2.07 (1.24, 3.46)^a^
	Year 2	0.24 (0.16, 0.35)^a^	0.92 (0.61, 1.39)
New alert		Reference	Reference
Views syndromic surveillance as a primary data source	Year 1	47.5 (4.2, 536)^a^	2.10 (0.26, 16.6)
	Year 2	3.86 (0.28, 52.9)	0.60 (0.12, 2.88)
Secondary		Reference	Reference

^a^Statistically significant result

## Discussion

### Main findings

The results of this study confirm the variation in approaches to syndromic surveillance as reported in previous studies [14–16]. In a survey of public health staff from United States state and local health agencies using syndromic surveillance systems, only 9% of local health departments were able to operate their syndromic surveillance system without state oversight [14–16]. These prior studies also highlight the impact of the lack of guidance regarding syndromic surveillance use, and how resource limitations contributed to the relative inability to create standard protocols to provide such direction [26]. Our study conveys a similar message, in that we observed strong differences in effect of the study’s intervention and how public health response patterns vary by population size served by health agencies and over time despite attempts to provide standardization by way of participatory intervention development.

Our intervention significantly decreased the number of responses in intervention versus control LPHAs in the first year but less in the second year of the study. In the first year, the intervention LPHAs were significantly less likely to report the findings to external agencies. This effect was not seen in year 2. This finding was unexpected and the reasons for the change are unclear. It could be due to a number of factors including; the introduction of new practices from the intervention protocol (an initial Hawthorne effect), the impact of the ice storm and disruption in year 1, or the higher number of alerts due to a more severe influenza season in year 2. In the post study interviews, LPHA staff reported no differences in their practice over time but they may not have been aware of minor subtle changes. More work is required to assess the reasons for this finding.

The interaction of the year of the study with the response to new versus sustained alerts appears to be mainly related to the process of “watchful waiting”. In the first year and with the exception of “watchful waiting”, LPHAs were more likely to respond to sustained alerts. In the post-study interviews, many staff reported that they would wait for a repeat alert before treating it seriously. They would implement “watchful waiting” for the first alert. In the second year, the responses were more likely to occur to first alerts but, on examination, most of this is driven by the outcome of “watchful waiting”. Using a direct test for statistical interaction, only the outcome of “all responses other than ‘watchful waiting’” had no statistical interaction to the year of the study (*p* = 0.86).

### Implications

The large confidence limits around all the logistic regression estimates for medium and small LPHAs in Table 4 raises the question about the minimum size of a health unit that can be served by syndromic surveillance, regardless of the provision of standardized supports such as algorithms and protocols. This was supported by the observations that small LPHAs, serving populations of less than 150,000, had limited resources to create response protocols and respond to syndromic alerts. The lower number of visits per day and reliance on one hospital created potentially statistically unstable data on which the syndromic alerts are based. This combination of lower staffing and unstable data generated challenges in interpreting the statistical and public health significance of aberrant events. These factors indicate the need to consider the minimum resources needed for the implementation of syndromic surveillance systems. A different approach is taken in England, where a standard protocol to assess all alerts is implemented by a central team [32]. Only alerts that meet selected criteria are passed on the local public health agencies, thereby limiting the amount of time and effort involved to complete such local investigations.

The failure to detect any outbreaks or consistently predict the onset of the influenza season supports the findings of Beuhler et al. [15]. Rather, syndromic surveillance is often listed as being useful in assessing the health impact of influenza on a community. Given the high number of alerts, the lower rates of response to these alerts by the LPHAs in the intervention group can be interpreted as them being more discriminating and thus more efficient.

The variation is the designation of alerts warranting public health action was unexpected, and was observed in both the intervention and control arm LPHAs. Given the mismatch between the reported actions in the free text fields and the answer to the question about the alert as warranting a public health response, we question how LPHA defined “assessment of public health significance”.

“Watchful waiting” was given as the response to one third of all syndromic alerts and it was most commonly associated with a new alert. The research team listed watchful waiting separately since, while LPHA staff considered it as part of situational awareness, it did not meet the study definition of effective use (Fig. 1). The implementation of standard protocols reduced the rates of “watchful waiting” (Table 4). Comments from participating LPHA staff in semi-structured interviews indicated that they would wait to see if an alert was sustained before taking an action other than “watchful waiting”. Such comments indicate a paradox of syndromic surveillance, where high sensitivity and real-time analysis to detect local aberrant events must be balanced with sensitive systems that generate a large number of false positive alerts. According to the findings of this study, it may increase the practice of waiting for repeated alerts or other changes in response protocols to order to meet resource constraints.

### Strengths and limitations

The strengths of the study are its prospective evaluation of syndromic surveillance in real-world public health settings for 16 months and spanning two respiratory disease seasons. The study also used an integrated knowledge translation approach where local public health communicable disease staff were actively involved in the intervention development, implementation, and evaluation process. In addition, the cluster-randomized experimental study design is a strong design for this type of evaluation. There was high retention with only one LPHA dropping out of the study. Evaluation of the intervention’s implementation and fidelity indicated that there were no evidence of contamination in that the LPHAs in the control arm reported no changes to their practices over the duration of the study.

The study has several limitations. The disruption to syndromic surveillance systems due to the ice storm over the winter in 2013 created challenges. In addition to requiring an extension of the study, it may also have sensitized the study participants to be increasingly aware of system disruptions for the rest of the study period. In particular, we note the higher number of alerts investigated for system disruptions in year 1. Regression models were repeated separately for year1 and year 2 in order to assess this potential bias and no major differences in the models were noted. A further limitation was the necessity to use repeated measures in the statistical models in order to account for variation between the participating LPHAs. This resulted in a decline in statistical power and possible explanation of non-significant results. Another challenge is that LPHA staff who reported in the logbooks may have “copied-and-pasted” their logbook entries due to resource limitations. As a result, it is possible that the logbook data may not reflect the actual actions taken. However, it was not feasible for the research team to observe and verify the validity of information captured in the logbook entries, aside from conducting semi-structured interviews to probe for findings that corroborate the results. A final limitation is that the study only examined respiratory and influenza-like illness syndromes in the Ontario, Canada public health context, and external generalizations beyond this setting may be limited.

## Conclusion

This study raises questions about the effectiveness of using standardized protocols to improve the performance of syndromic surveillance in a decentralized public health system. Despite efforts to create standardized protocols and engage public health agencies in the process, no significant differences in the effective use of syndromic alerts were observed. The only significant change was a reduction in the practice of watchful waiting. The study also raises questions about the minimum capacity of an agency and minimum population size that are required for the effective use of syndromic surveillance.

## Appendix

### Development of aberrant event detection algorithms

To determine the optimal aberrant event detection method to implement as the study intervention algorithm in LPHAs, several statistical methods and their relative sensitivity and specificity were evaluated. In the first step, we used regression models were used to account for seasonality of day of the week effects, using historical syndromic surveillance data received from participating Ontario LPHAs jurisdictions for the time period of September 1, 2010 to August 31, 2013. To account for varying daily emergency department visit volumes each day, the total count of these visits was used as an offset in the appropriate regression models. Differences between observed number of events and expected number of events estimated by regression models (the residuals) were the main outcome from these models.

We then applied methods of statistical process control to the residuals from these regression models. The statistical process control methods used in this study included the generalized version of the Early Aberration Reporting System (EARS) [A1], the cumulative sum control chart method or CUSUM [A2], and unweighted moving average (MA) and exponential weighted moving average methods (EMA) [A3].

For the EARS method, instead of a weighting each observation equally with fixed threshold (h) and correction parameter (k), the means and standard deviations are calculated with weighted observations and h and k were estimated from data. For MA, means of a short moving window (length S, prospective data) and a long moving window (length L, historical data) are compared with a threshold of h. While in EMA, the means and standard deviations are calculated with exponential weights, weighting later observations more than earlier observations.

Optimization of all Cusum and two moving average models were done using simulated data. Based on historical trends and using Monte Carlo methods, increased risks of 10, 15 and 20% were randomly added over a two week period. Regression models and each statistical process control method were repeated run in order to estimate average run lengths (ARLs) and tuning parameters for each algorithm. The process was repeated until the parameters converged.

For the emergency department data, a linear regression model performed best. In order to assess which statistical process control approach (EARS, CUSUM, MA, or EMA) was best, the models for each algorithm were assessed by examining the percent of simulated outbreaks detected and the average run length to detect the outbreak (ARL1). For these calculations, we used a fixed average run length to have no outbreaks (ARL0).

In order to evaluate the performance of these statistical process control methods (EARS, CUSUM, MA, or EMA), data were simulated with similar characteristics of the historical data with various magnitudes of rises (10, 15 and 20%) in a 2-week period. In the simulations, ARL0 (the average time between two (false) alerts when the system is in control, i.e. no aberrations) and the threshold (h) were fixed at preselected values. For example, for the Cusum model, the ARL0 was predetermined to be 150 days, considered the minimum time between influenza seasons. The measures of ARL1 (average time the algorithm takes to alert when there are aberrations in the system) and k (or S/L for MA and EMA) were estimated and compared. By selecting the algorithms with the highest percent of outbreaks detected and lowest ARL1, nine potential detection algorithms were selected for further consideration by end-users – three for each of Cusum, moving average and weighted moving average consisting of methods with high sensitivity, low specificity; balanced sensitivity and specificity; low sensitivity and high specificity. A tenth model (EARS) was also included.

The results of these 10 models were plotted and discussed at a consensus meeting of the intervention LPHAs. Using a nominal group technique {reference}, the optional algorithm (in terms of sensitivity and specificity) and the resultant parameters were chosen.

The successful model was as follows: Let Y_t_ and E_t_ be the observed count and population size for day t, then we model the ratio Y_t_/E_t_ as outcome of a linear regression

1 $$ \begin{array}{l}{\mathbf{Y}}_{\mathbf{t}}/{\mathbf{E}}_{\mathbf{t}}\sim \mathrm{N}\left({\upmu}_{\mathrm{t}},{\upsigma}^2\right)\\ {}\kern1em {\upmu}_{\mathrm{t}} = {\mathrm{X}}_{\mathrm{t}}\upbeta +{\upepsilon}_{\mathrm{t}}\end{array} $$

where μ_t_ is the modelled expected count for day t, which depends on covariates X_t_ that include seasonality and day of week effects and є_t_ follows a normal distribution with mean 0 and variance σ^2^.

The CUSUM model with a high specificity was the optimal detection method. The system alerts when CUSUM exceeds a threshold value (h):

2 $$ {\mathrm{S}}_{\mathrm{t}}= \max \left[0,{\mathrm{S}}_{\mathrm{t}\hbox{-} 1}+{\upepsilon}_{\mathrm{t}}\ \hbox{-} \mathrm{k}\right] > \mathrm{h} $$

Formula 2 is a classic CUSUM algorithm where a CUSUM value (S_t_) is non negative and S_0_ = 0, h is an alert trigging threshold and k is a correction factor that brings the CUSUM value closer to 0 at each run. These two parameters together determine the two Average Run Lengths (ARL0 and ARL1) concepts in process control literature. For the algorithm, h was set to 2 and k was set to 1.12 to achieve a ARL0 of 150 days and ARL1 of 7 days. A flag is generated when CUSUM value exceeds threshold h on 2 consecutive days. During the study, the CUSUM value was reset to zero after two flags on consecutive days was reached.

## Abbreviations

LPHA: Local Public Health Agency (Public Health Unit)
SSES: Syndromic surveillance evaluation study
